# Correlation between Skeletal Maturation and Developmental Stages of Canines and Second Molars among Iranian Population

**DOI:** 10.30476/DENTJODS.2021.87505.1266

**Published:** 2022-06

**Authors:** Masume Firouzinia, Soraya Khafri, Maysam Mirzaie, Farida Abesi, Mahtab Hamzeh

**Affiliations:** 1 Student Research Committee, Babol University of Medical Sciences, Babol, Iran; 2 Dept. of Biostatistics and Epidemiology, Faculty of Medicine, Babol University of Medical Sciences, Babol, Iran; 3 Dental Materials Research Center, Health Research Institute, Babol University of Medical Sciences, Babol, Iran; 4 4Dept. of Oral & Maxillofacial Radiology, School of Dentistry, Babol University of Medical Sciences, Babol, Iran; 5 5Dept. of Pediatric Dentistry, Babol University of Medical Sciences, Babol, Iran

**Keywords:** Teeth Calcification, Cervical Vertebrae, Panoramic Radiography, Cephalometry

## Abstract

**Statement of the Problem:**

Growth assessment has become an important issue in many medical and dental fields. Determining the stages of dental development and skeletal maturation are essential methods in evaluation of growth phases.

**Purpose:**

This study aimed to assess the relationship between developmental stages of maxillary and mandibular canines and second molars by Nolla’s method and skeletal maturation stages by cervical vertebral maturation. In addition, diagnostic performances of dental developmental stages were evaluated to identify growth phases.

**Materials and Method:**

In this descriptive-analytical study, 201 digital panoramic and lateral cephalometry of children referred to the Orthodontic Department of Babol Dental School (8 to 15 years) were examined. The stages of dental development were determined by Nolla’s method, and the stages of skeletal development were determined by cervical vertebral maturation stages (CVMs). Positive likelihood ratio (LHR+) was used to determine the diagnostic performances to identify growth phases. The Kendall's Tau-b correlation coefficient was used to measure the association between the CVM stages and dental calcification stages. In this study, *p* ≤ 0.05 was considered significant.

**Results:**

The relationship between dental development and skeletal maturation in different teeth was significant (*p* ≤ 0.05). Kendall's Tau-b correlation coefficient between the stages of dental development and skeletal maturity in girls ranged from 0.578 - 0.634 and in boys ranged from 0.588 - 0.655. The right second molar in maxilla presented the highest correlation coefficient. The right and left second molars in maxilla and left second molar in mandible in stage 5 had the highest LHR+ to identify the pre-pubertal growth phase.

**Conclusion:**

Despite the high correlation coefficient between dental development and skeletal maturity, the LHR+ for determining growth stages in only a small number of teeth was more than 10.

## Introduction

Recently, the optimal treatment schedule for achieving the most desirable response with the least chance of treatment failure has been considered by both researchers and physicians. Studies have shown that successful treatment of skeletal disharmonies can be predicted by initiating the individual pubertal growth spurt [ 1 ]. Knowledge of the stage of maturation has also been well established in forensic sciences and syndrome identification [ 2 ].

Biological or physiological maturity is introduced according to the diversity of children's development at the same chronological age. Physiological maturity is determined by the different biological indicators of maturity [ 3 ]. Sexual maturation individuality, chronologic age, dental development, height, weight, and skeletal development are a number of the most frequent maturity values employed to identify different growth stages [ 4 ].

There are three reliable indicators of individual skeletal maturity, including increase in stature height, skeletal maturation of the hand and wrist, and changes in the morphology of the cervical vertebrae. The use of the first two indicators in everyday clinical practice currently is limited [ 5 ]. In contrast, the cervical vertebral maturation stages (CVMs) method, which was proposed by Baccetti *et al*. [ 6 ], is mostly accepted at present [ 7 ]. CVMs involves all important phases in craniofacial growth during adolescence and young adulthood, is applicable for both genders, and entails no additional X-Ray exposure beyond the regular lateral cephalometric radiography [ 8 ].

Tooth calcification and tooth eruption are two methods of evaluating tooth development. Tooth eruption is affected by malnutrition, the early loss of deciduous teeth, dental caries, and crowding; hence, it is a variable and discontinuous parameter [ 9 ]. In addition, tooth eruption time cannot be applied between 3 to 6 years or after the age of 13; therefore, it is thought that tooth formation is a more reliable variable [ 10 ]. There are some methods to evaluate dental development by using calcification stage. The most common method in clinical training and performance is proposed by Nolla *et al*. [ 11 ]. This technique can assess the mineralization of each tooth of the maxillary and mandibular arch [ 12 ]. Some studies have shown that this method is highly reliable in different populations [ 13 - 16 ]. Caro and Contreras [ 17 ] reported that Nolla's method offers more accurate results than other methods for determining dental age. Björk and Helm [ 19 ] also reported that the maximum growth maturation in girls and boys occurs at about 12 and 14 years, respectively. 

As dentistry progresses straightforward and time-saving, and also panoramic radiographs are commonly used in most dental clinics, these radiographs can be used as a suitable alternative for hand-wrist radiography to evaluate individual maturity [ 20 ]. The relationship between dental development by Demirjian method and CVMs methods in Iran has been assessed [ 20 - 21 ].

Many studies show that Demirjian's dental development method could have been clinically useful as a skeletal maturity index, and the calcification of the second mandibular molars and mandibular canines showed the highest correlation with skeletal maturation [ 12 , 22 - 25 ]. 

This study, with the help of the CVMs method and Nolla’s method, was designed to evaluate the relationship between developmental stages of maxillary and mandibular canines and second molars and skeletal maturation by the morphology of cervical vertebrae in a population of Iran. 

## Materials and Method

In this cross-sectional study, 201 digital panoramic and lateral cephalometric radiographs of patients referred to the Orthodontics Department of Babol University of Medical Sciences (89 males and 112 females, the age range of 8-15 years) were examined. This study was approved by the Ethics Committee of Babol University of Medical Sciences (ethical code: IR.MUBABOL. REC.1398.023). The inclusion criteria were (1) Iranian patients aged 8 to 15 years, (2) high clarity and good contrast of lateral cephalometric and panoramic radiographs, (3) no missing, extraction, or anomalies in dentition, (4) no positive dental history of previous orthodontic treatment, trauma, or surgery in the neck or dentofacial region, (5) no maxillofacial defects such as cleft lip and palate, (6) no systemic disease that could influence the growth and development such as hormonal disease, and finally (7) absence of any cervical vertebral anomalies. After sample selection, by subtracting the date of birth from the date of radiography, the chronological age at the time of radiography was obtained. Radiographic images were examined visually in the same condition and simultaneously by two observers who were pediatric dentist and orthodontist, in a completely dark room on a 17-inch screen (Samsung syn master DFX 1793), without time constraints, and a single opinion. In case of disagreement, the third observer (maxillofacial radiologist) has assisted for final verdict.

**Figure 1:**
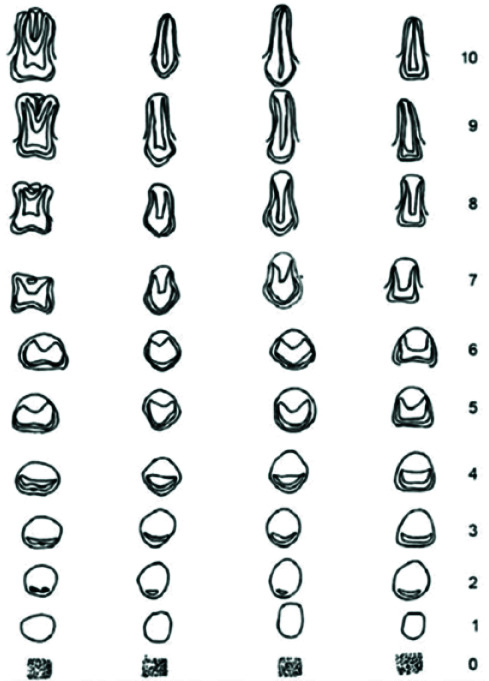
Tooth calcification stages according to Nolla’s method [11]. Stage 0: Absence of crypt, Stage 1: Presence of crypt, Stage 2: Initial calcification, Stage 3: One-third of crown completed, Stage 4: Two-third of crown completed, Stage 5: Crown almost completed, Stage 6: Crown completed, Stage 7: One-third of root completed, Stage 8: Two-third of root completed, Stage 9: Root almost complete; open apex, Stage10: Apical foramen of root closed.

**Figure 2:**
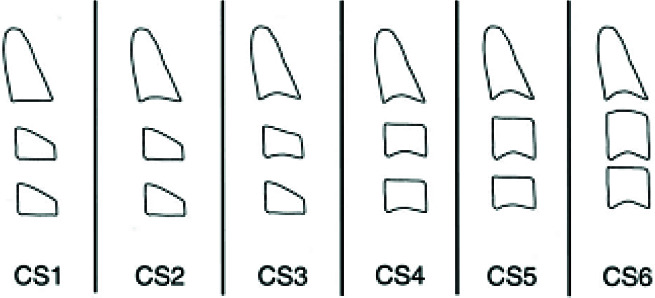
Cervical Vertebral Maturation Stages (CVMs) Method (Baccetti *et al*.) [ 6 ]

Dental stages of permanent canines and second molars on the left and right side of maxilla and mandible were determined using panoramic radiographs. These stages were estimated according to Nolla’s method [ 11 ], which divides dental calcification into ten stages (Figure 1). Skeletal stages were verified by using lateral cephalometric radiographs. These stages were assessed regarding the CVMs method suggested by Baccetti *et al*. [ 6 ]. The morphology of the second, third, and fourth cervical vertebrae are analyzed in this method, and the CVM id classiﬁed into six stages (Figure 2). The characteristics of the stages are described in Table 1. In this study, the growth phases were divided into (CVMs 1-CVMs 2) as pre-pubertal, (CVMs 3-CVMs 4) as pubertal, and (CVMs 5-CVMs 6) as post-pubertal [ 9 ].

**Table 1:** Characteristics of cervical vertebral maturation stages (CVMs) by Baccetti *et al*. [ 6 ]

CVMs	The lower border of c2	The lower border of c3	The lower border of c4	The shape of the body c3	The shape of the body c4	Time
CVMs1	Flat	Flat	Flat	Trapezoid	Trapezoid	A peak in mandibular growth would occur on average 2 years after this stage
CVMs2	Concave	Flat	Flat	Trapezoid	Trapezoid	A peak in mandibular growth would occur on average 1 year after this stage
CVMs3	Concave	Concave	Flat	Trapezoid/Rectangular horizontal	Trapezoid/ Rectangular horizontal	A peak in mandibular growth would occur during the year after this stage
CVMs4	Concave	Concave	Concave	Rectangular horizontal	Rectangular horizontal	A peak in mandibular growth has occurred between 1 or 2 years after this stage
CVMs5	Concave	Concave	Concave	At least one of C3 or C4 of the bodies is square	At least one of C3 or C4 of the bodies is square	A peak in mandibular growth has ended at least 1 year before this stage
CVMs6	Concave	Concave	Concave	Rectangular vertical	Rectangular vertical	A peak in mandibular growth has ended at least 2 years before this stage

SPSS software (v.20) was used for statistical analysis; ANOVA test was used to compare the mean chronological age at different stages of CVM and teeth development in general and by gender. Due to the inequality of variances, the Games-Howell post-hoc test was used to compare the mean age between two stages of CVMs. T-Test was used to compare mean age at different CVMs and dental development between girls and boys. Kendall's Tau-b correlation coefficient was used for analyzing the correlations between CVMs and chronological age and CVMs and teeth calcification.

Probability values of *p* ≤ 0.05 were considered statically significant. Diagnostic performances were evaluated based on identifying the growth phases using LHR+ LHR+ estimates how the dental maturation stage changes the chances of having certain CVMs. LHR+ more than 1 indicates that the test result is related to certain CVMs, while LHR+ less than 0.1 makes it almost impossible to have certain CVMs. LHR+10 or more than 10 was considered for evaluation of satisfactory reliability of each dental development stage for identifying each growth phase [ 26 ]. Therefore, in this study, LHR+ was considered 10 or more than 10.

## Results

In this study, 201 digital panoramic and lateral cephalometric radiographs of 8 to 15 years old patients were examined; of whom 112 were girls (55% with an average age of 10.19) and 89 were boys (45% with an average age of 10.43). The lowest and highest frequencies were in CVMs2 and CVMs3 and the minimum and maximum ages were 8 and 15 years, respectively. Table 2 shows the frequency of calcification of maxillary and mandibular canines and second molars at each CVMs.

**Table 2:** The frequency of calcification of maxillary and mandibular canines and second molars at each cervical vertebral maturation stages (CVMs), M (male), F(female)

CVMs	Dental Stage	Tooth 13		Tooth 23		Tooth 33		Tooth 43		Tooth 17		Tooth 27		Tooth 37		Tooth 47	
		M	F	M	F	M	F	M	F	M	F	M	F	M	F	M	F
CVMs 2	5	-	-	-	-	-	-	-	-	3	1	3	1	2	1	3	1
	6	2	-	2	-	-	-	-	-	4	2	4	2	4	1	3	1
	7	5	2	4	2	6	1	5	2	-	2	-	2	1	3	1	3
	8	-	3	1	3	1	4	2	3	-	-	-	-	-	-	-	-
CVMs 3	6	-	-	-	-	-	-	-	-	28	27	28	25	17	21	17	17
	7	24	13	23	13	17	13	19	14	12	19	12	22	19	22	19	26
	8	20	33	21	33	26	33	24	32	3	2	3	1	7	5	7	5
	9	1	2	1	2	2	1	2	1	2	-	2	-	2	-	2	-
	10	-	-	-	-	-	1	-	1	-	-	-	-	-	-	-	-
CVMs 4	5	-	-	-	-	-	-	-	-	-	1	-	1	-	-	-	-
	6	-	-	-	-	-	-	-	-	4	3	4	4	-	1	-	2
	7	5	1	4	-	2	-	2	1	8	13	9	12	12	13	13	13
	8	16	24	17	25	15	23	15	24	8	13	7	13	7	15	8	15
	9	6	8	5	8	8	6	10	5	7	6	7	6	8	7	6	6
	10	-	3	1	3	2	7	-	6	-	-	-	-	-	-	-	-
CVMs 5	6	-	-	-	-	-	-	-	-	-	1	2	4	-	1	-	1
	7	-	-	-	-	-	-	-	-	-	1	3	7	1	1	1	1
	8	2	5	2	5	2	4	2	4	2	8	5	12	1	8	1	8
	9	4	11	4	12	3	7	3	7	7	11	7	11	7	13	6	13
	10	4	7	4	6	5	12	5	12	1	2	1	2	1	-	2	-

Kendall's Tau-b correlation coefficients between chronological age and CVMs was 0.513 in general, 0.547 in girls, and 0.517 in boys; which was statistically significant (*p* ≤ 0.05). 

The mean chronological age was significantly different in all stages of CVM (*p* ≤ 0.05). The mean age difference between the two stages of CVM was generally statistically significant (*p* ≤ 0.05); however, there was no significant difference between cs4 and cs5 in girls, cs2, cs3, cs4 and cs5 in boys (*p* > 0.05). 

The relationship between dental development stages and mean chronological age in general and by gender was statistically significant; as the dental development stages in different teeth increased, the average chronological age increased significantly too (*p* ≤ 0.05). The mean chronological age in each dental stage in boys was higher than girls. Moreover, the results of T-Test to compare the mean of chronological age in different stages of dental development between girls and boys showed that this difference was not significant (*p* > 0.05).

Table 3 shows Kendall's Tau-b correlation coefficients between CVMs and the stages of tooth development in different teeth. In this study, the teeth 13, 23, 43 in stage 10 of dental development had LHR+ above 10 to detect the post-puberty phase. The teeth 17, 27 and 47 in stage 5 of dental development had LHR+ above 10 to detect the pre-puberty phase. Table 4 presents sensitivity, specificity, positive predictive value (PPV) and LHR+ in teeth with LHR+ greater than 10.

**Table 3:** Kendall’s Tau-b correlation coefficients between cervical vertebral maturation stages (CVMs) and the stages of tooth development in different teeth

Gender Tooth	Female	Male	General
13	0.579 (<0.001)	0.607 (<0.001)	0.594 (<0.001)
23	0.592 (<0.001)	0.591 (<0.001)	0.596 (<0.001)
33	0.589 (<0.001)	0.598 (<0.001)	0.599 (<0.001)
43	0.589 (<0.001)	0.588 (<0.001)	0.595 (<0.001)
17	0.634 (<0.001)	0.655 (<0.001)	0.639 (<0.001)
27	0.625 (<0.001)	0.651 (<0.001)	0.631 (<0.001)
37	0.627 (<0.001)	0.610 (<0.001)	0.615 (<0.001)
47	0.609 (<0.001)	0.608 (<0.001)	0.605 (<0.001)

The numbers show Kendall’s tau-b correlation coefficients (*p* Value)

**Table 4:** Sensitivity, specificity, PPV and LHR+ in the teeth with LHR+ greater than 10

Diagnostic variable	Diagnostic tests	Sensitivity (95% CI)	Specificity (95% CI)	PPV (95% CI)	LHR+ (95% CI)
Stage 10 of tooth 13 to detect the post-puberty phase		33% (17-49)	98% (96-100)	79% (57-100)	18.67 (5.31-63.28)
Stage 10 of tooth 23 to detect the post-puberty phase		30% (15-46)	98% (95-100)	71% (48-95)	12.73 (4.25-38.15)
Stage 10 of tooth 43 to detect the post-puberty phase		52% (34-69)	96% (93-99)	71% (53-89)	12.36 (5.57-27.44)
Stage 5 of tooth 17 to detect the pre-puberty phase		33% (7-60)	99% (98-100)	80% (45-100)	63 (7.26-520.80)
Stage 5 of tooth 27 to detect the pre-puberty phase		33% (7-60)	99% (98-100)	80% (45-100)	63 (7.26-520.80)
Stage 10 of tooth 47 to detect the post-puberty phase		33% (7-60)	99% (98-100)	80% (45-100)	63 (7.26-520.80)

LHR+: Positive Likelihood Ratio; PPV: Positive Predictive Value; 95% cl: 95% Confidence Interval

## Discussion

Identifying a rapid growth period is significantly effective in the skeletal treatment in orthodontic patients. Assessment of growth phases is also an integral part of the diagnosis and treatment program for children [ 27 ]. Over the years, various methods have been developed to determine growth phases, including wrist radiography, elbow radiography and cervical vertebral morphology [ 12 ].

The relationship between tooth calcification and CVMs is debatable. Some studies confirm this relation, while some researchers have reported a weak correlation between them. Moreover, the effect of racial changes and different methods has been reported to determine the stages of dental development and skeletal maturation [ 20 ].

In this study, there was a relatively high correlation coefficient between chronological age and skeletal maturity in general and concerning the gender, being higher in female participants. These results were in agreement with the study of Abesi *et al*. [ 28 ] and Baidas *et al*. [ 29 ], while in the studies of Alkhal *et al*. [ 30 ], Uysal *et al*. [ 31 ] and Stiehl *et al*. [ 32 ], relatively lower correlation between the chronological age and CVMs for boys and girls were reported.

The differences in the results of different studies can be due to differences in race, geographical environment, selected age groups, sample size, and sample selection method. In this study, the high correlation between the chronological age and the CVMs showed that with the increase of chronological age, CVMs has increased either; however, wide variations in chronological age for different stages of the cervical vertebrae maturity showed that chronological age is not an accurate indicator for determining maturity stages. These results were in agreement with the study of Abesi *et al*. [ 28 ], Baidas *et al*. [ 29 ] and Alkhal *et al*. [ 30 ]. Also, studies by Baidas *et al*. [ 29 ], Alkhal *et al*. [ 30 ] and Tiziano Baccetti *et al*. [ 33 ] showed that chronological age is not a reliable in dicator for assessing maturity stages.

The mean age difference between the two stages of cervical maturation was generally significant (*p* ≤ 0.05); but there was no significant difference between the levels of CVMs4 and CVMs5 in girls, CVMs2 and CVMs3 and CVMs4 and CVMs5 in boys (*p* > 0.05). The mean chronological age at each CVMs was higher in boys than in girls, but this difference was not significant in each CVMs. As reported in the studies of Tiziano Baccetti *et al*. [ 33 ], Abesi *et al*. [ 28 ], Baidas *et al*. [ 29 ] and Nemati *et al*. [ 9 ], in the present study, girls were ahead of boys in skeletal maturity and skeletal maturity in girls had begun earlier.

In this study, dental development in girls occurred earlier than boys; which was in agreement with the study of Hägg and Taranger [ 18 ] and Fishman [ 34 ]. In addition, like the study of Nolla *et al*. [ 11 ], Sachan *et al*. [ 25 ], this difference was not significant in any of the different stages of dental development (*p* > 0.05).

In this study, there was a significant relationship between CVMs and dental development by the Nolla’s method. In both girls and boys, the highest Kendall’s Tau-b correlation coefficient was for tooth 17, and the lowest Kendall correlation coefficient was for tooth 13 in girls and tooth 43 in boys. In the studies of Nemati *et al*. [ 9 ], Trakinienė *et al*. [ 35 ] and Uysal *et al*. [ 31 ], tooth 7 had the highest correlation coefficient between CVMs and dental development.

The correlation coefficient was higher in boys than in girls. The present study showed that despite the correlation between different stages of dental development and maturation of cervical vertebrae, overall diagnostic performance of using the teeth calcification stages to identify growth stages in many cases has low LHR+. In this study, Teeth13, 23, 43 in stage 10 of dental development, had LHR+ above 10 to detect post-pubertal phase. Teeth 17, 27 and 47 in stage 5 of dental development, had LHR+ above 10 to detect the prepuberty phase.

In a study conducted by Kamal *et al*. [ 12 ], tooth 33 in stages 9 and 10 of dental development showed CVMs2 and CVMs3 and tooth 37 in 8-10 dental development stages represented CVMs3. In a study conducted by Al-Balbeesi *et al*. [ 22 ], tooth 33 in stages 9 and 10 of dental development represented pubertal phase. 

In the study of Günen Yılmaz *et al*. [ 36 ], tooth 37 in stage 8-7 of dental development represented the prepubertal phase, and stage 10 of dental development represented the post-pubertal phase.

In a study conducted by Sachan *et al*. [ 24 ], tooth 33 in the stage between 8 and 9 of dental development indicated the early stages of developmental maturation.

The various results expressed in the studies may be related to different methods of data collection, different methods of determining the stages of skeletal or dental maturation, geographical environment, selected age groups, racial changes, and size of the sample.

There were some limitations in the present study which would be considered while interpreting the results. The radiographs were taken by different devices which may affect the quality of radiographs and the diagnosis of developmental stages. On the other hand, the study samples were predominantly from one province of Iran and the results cannot be easily generalized to Iranian population. Thus, it is recommended that the sample size should be larger and from all provinces of Iran to confirm the present results in further extensive studies.

## Conclusion

This study showed that in this population, the relationship between the tooth calcification by Nolla’s method and the maturation of cervical vertebrae was significant. Tooth 17 presented the highest relationship in both genders. Despite the high correlation coefficient between the dental calcification stages and the CVMs, diagnostic ability of using the teeth calcification stages to identify growth phases would be limited. Although Calcification stages of teeth 13, 23 and 43 in stage 10 had a satisfactory diagnostic value for predicting post-pubertal phase, and teeth 17, 27 and 47 in stage 5 of dental development had a satisfactory diagnostic value for predicting prepubertal phase, but the calcification stages were not reliable for detecting pubertal phase.

## Conflict of Interest

The authors report no conflict of interest.
